# PDIA3 driven STAT3/PD-1 signaling promotes M2 TAM polarization and aggravates colorectal cancer progression

**DOI:** 10.18632/aging.205847

**Published:** 2024-05-17

**Authors:** Jianchun Fan, Likun Wang, Chunze Zhang, Xueliang Wu, Lei Han, Xiaoyu Zhang, Shuquan Gao, Jun Xue, Qi Zhang

**Affiliations:** 1 Graduate School, Hebei North University, Zhangjiakou 075000, China; 2 Department of Ultrasound Medicine, The First Affiliated Hospital of Hebei North University, Zhangjiakou 075000, China; 3 Department of Anus and Intestine Surgery, Tianjin People's Hospital, Tianjin 300122, China; 4 Department of General Surgery, The First Affiliated Hospital of Hebei North University, Zhangjiakou 075000, China; 5 Institute of Tumor, The First Affiliated Hospital of Hebei North University, Zhangjiakou 075000, China; 6 Integrated Chinese and Western Medicine Hospital, Tianjin University, Tianjin 300100, China

**Keywords:** cancer microecology, immunological subterfuge, neoplastic therapy, colorectal neoplasm, PDIA3

## Abstract

Objective: This inquiry endeavors to delineate the influence of PDIA3 on tumor-associated macrophages within the realm of colorectal malignancies, whilst elucidating the intrinsic biochemical pathways.

Method: Leveraging bioinformatics, we scrutinized the symbiosis between PDIA3, STAT3, and CD274. A xenograft model in immunodeficient murine served to assess PDIA3's impact on colorectal carcinogenesis. Further, Western blot analysis quantified the protein expression of PDIA3, p-STAT3, PD-1, XBP-1, assorted enzymes, and IL-6. Moreover, *in vitro* assays gauged SW480 cellular dynamics inclusive of migration, invasive potential, and proliferation.

Results: Bioinformatics exploration exposed PDIA3's elevated presence in diverse cancers, with a marked expression in colorectal cancer, as per TCGA and GEO repositories. Correlative studies showed PDIA3 positively aligning with STAT3 and CD274, the latter also associated with monocyte-derived macrophages. Comparative analysis of colorectal neoplasms and normal colon samples unveiled heightened levels of PDIA3 markers which, when overexpressed in SW480 cells, escalated tumorigenicity and oncogenic behaviors, with a noted decrease upon PD-1 monoclonal antibody intervention.

Conclusions: PDIA3 augments the M2 polarization of tumor-associated macrophages via modulation of the STAT3/PD-1 cascade, thus invigorating the tumorous proliferation and dissemination in colorectal cancer. Such revelations position PDIA3 as an auspicious target for PD-1 blockade therapeutics, offering a promising foundation for rectifying colorectal carcinoma.

## INTRODUCTION

Colorectal cancer (CRC), a profoundly prevalent and pernicious neoplasm, garners a ranking as the third most common cancer globally [1–3]. A myriad of risk factors coalesces to predispose individuals to CRC, encompassing inherited genetic predispositions, environmental influences, lifestyle choices, dietary [4, 5].

In the realm of clinical therapeutics for colorectal cancer, a multifaceted approach is often embraced, integrating surgical intervention, chemotherapy, radiotherapy, molecular-targeted therapy, immunotherapy, and palliative care measures. A burgeoning frontier within the therapeutic landscape is tumor immunotherapy, which promises a novel paradigm in oncological treatment by modulating the immunologic milieu to thwart tumor proliferation and dissemination. Within the immune system’s arsenal, macrophages stand as sentinel effector cells, exhibiting a pivotal influence on tumoral dynamics. These cells differentiate into divergent phenotypes: the M1 macrophages [6], which conjure anti-inflammatory and anti-neoplastic properties, and the M2 macrophages [7], which elicit pro-inflammatory and immunosuppressive responses conducive to tumor facilitation via immune evasion and oncogenic stimulation. Tumor-associated macrophages (TAMs) represent a quintessential component of the tumor’s immune context, orchestrating cancer progression, invasion, and metastatic spread through a symphony of cytokine and chemokine release alongside interaction with inflammatory pathways. Notably, a slew of research evidences an association between the preponderance of TAMs and adverse prognostications across a spectrum of solid malignancies. Contrastingly, in the specific theater of CRC, the strategic reprogramming of TAM polarization might potentiate the efficacy of immunotherapeutic interventions [8].

PDIA3, a distinguished constituent of the protein disulfide isomerase (PDI) family, executes a cardinal role in facilitating the formation, redox modulation, and isomerization of protein disulfide bonds. Its primary locus of activity is situated within the endoplasmic reticulum (ER), though it also garners a presence within cellular membranes and in the extracellular matrix [9, 10]. PDIA3’s relationship with cancerous transformation and progression are both intimate and insidious, with heightened expression levels detected in a gamut of malignancies, including diffuse glioma [11], clear cell renal cell carcinoma [12], hepatocellular carcinoma (HCC) [13], breast cancer [14], cervical cancer [15], and gastric cancer [16], an overexpression which traditionally signals a grim prognosis. In glioblastoma cellular models, PDIA3 has shown a suppressive impact on M2 macrophage polarization and pro-inflammatory cytokine synthesis [17]. Moreover, it drives the onward march of HCC through the STAT3 signaling cascade [18] and, as expounded by illustrious researchers, has been pinpointed as a promising target for therapeutic endeavors against breast cancer cells [14]. An elevation of PDIA3 expression has been discerned within subsets of CRC-afflicted patients, and intriguingly, the occurrence of PDIA3 autoantibodies may serve as an auspicious marker for the fine-tuning of vaccine preselection strategies [19]. Comparative analyses reveal overexpression of PDIA3 in CRC tissues vis-à-vis adjacent non-malignant tissues, with a parallel upregulation noted in CRC cell lines. Experimental gene silencing of PDIA3 via siRNA in SW480 cells induces profound subcellular morphological alterations, curtails cellular proliferation, and accentuates apoptotic processes [20]. Recent scientific discoveries have shed light on PDIA3's influential role in modulating the behavior of tumor-associated macrophages, in particular, its regulatory capacity over the STAT3/PD-1 (signal transducer and activator of transcription 3/programmed cell death protein 1) signaling axis [21]. PD-1, an inhibitory checkpoint molecule within the immune system, plays a cardinal role in fostering immune tolerance, thus stymieing the body’s inherent anti-tumor immune responses. Conversely, STAT3 stands prominent as a critical transcription factor implicated in immune function modulation and inflammatory response orchestration.

Our current conjectures categorically suggest that PDIA3 might considerably modulate the M2 polarization within tumor-associated macrophages, thereby augmenting the tumorigenic capacity of colorectal cancer cells via manipulation of the STAT3/PD-1 signaling pathway. Under this, we have scrutinized this explanatory discourse to unveil novel therapeutic targets for colorectal cancer. Nevertheless, we harbor reservations regarding whether such proclamations might be unduly optimistic. Is the putative role of PDIA3 in the M2 polarization of tumor-associated macrophages overly magnified? Does the STAT3/PD-1 signaling axis truly occupy a pivotal role in enhancing the oncogenic potential of colorectal cancer cells? Moreover, are novel therapeutic targets genuinely efficacious, or do they merely seem potent in theory? We necessitate more compelling evidence to bolster these assertions, as opposed to merely acquiescing to these suppositions.

## RESULTS

### In-depth bioinformatics scrutiny illuminates the pronounced upregulation of PDIA3 in colorectal carcinoma, concomitant with its affirmative association with STAT3, CD274, and monocytic/macrophage markers

**Figure 1:**
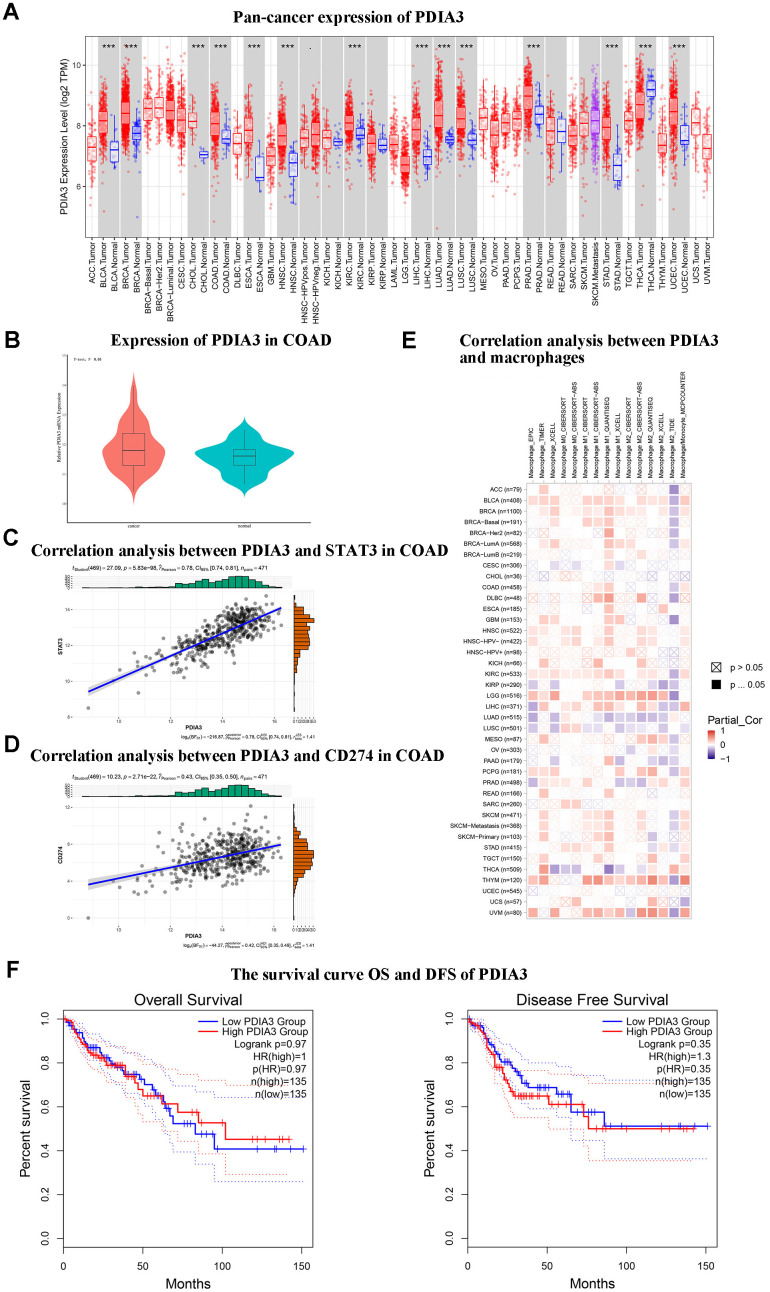
**Bioinformatic analyses suggest that PDIA3 exhibits notably elevated expression in colorectal cancer (CRC) and demonstrates significant associative upregulation with STAT3, CD274, and monocyte/macrophage levels.** (**A**) Widespread expression of PDIA3 across multiple cancer types. Statistical comparison via T-test, P<0.001. (**B**) Differential expression of PDIA3 within colorectal adenocarcinoma (COAD). Statistical comparison via T-test, P<0.05. (**C**) Evaluative correlation between PDIA3 and STAT3 expressions in COAD. Correlation coefficient (R) = 0.78, P=0.85E-98. (**D**) Evaluative correlation between PDIA3 and CD274 expressions in COAD. Correlation coefficient (R) = 0.43, P=2.71E-22. (**E**) Analysis of the correlation between PDIA3 and macrophages in pan cancer. Statistical comparison via T-test, P<0.05. (**F**) Survival analysis depicting Overall Survival (OS) (Log-rank P=0.97) and Disease-Free Survival (DFS) (Log-rank P=0.35) as influenced by PDIA3 expression in COAD.

An initial survey utilizing the TIMER2.0 databank to assess pan-cancer expression patterns unveiled a marked overexpression of PDIA3 in colorectal carcinoma (Figure 1A). Subsequent verification via the GEO database (GSE20916) corroborated this enhanced PDIA3 expression within colorectal malignancies (Figure 1B). Employing the R package “corrplot” we established a positive interrelation between PDIA3 and the STAT3 gene, as well as between PDIA3 and CD274 (Figure 1C, 1D). Moreover, analyses predicated on TIMER2.0 insinuated a positive correlation between CD274 and the monocytes/macrophages axis, alluding to their potential complicity in the colorectal cancer spectrum (Figure 1E). Survival analysis depicting Overall Survival (OS)and Disease-Free Survival (DFS) as influenced by PDIA3 expression in COAD (Figure 1F). These findings suggest that PDIA3 may influence the pathogenesis of colorectal cancer by modulating mononuclear macrophage differentiation, thus warranting further investigation into the interconnectivity of PDIA3 with macrophage polarization proteins and their signaling pathways.

### Pronounced PDIA3 protein prevalence in colorectal carcinoma specimens

**Figure 2:**
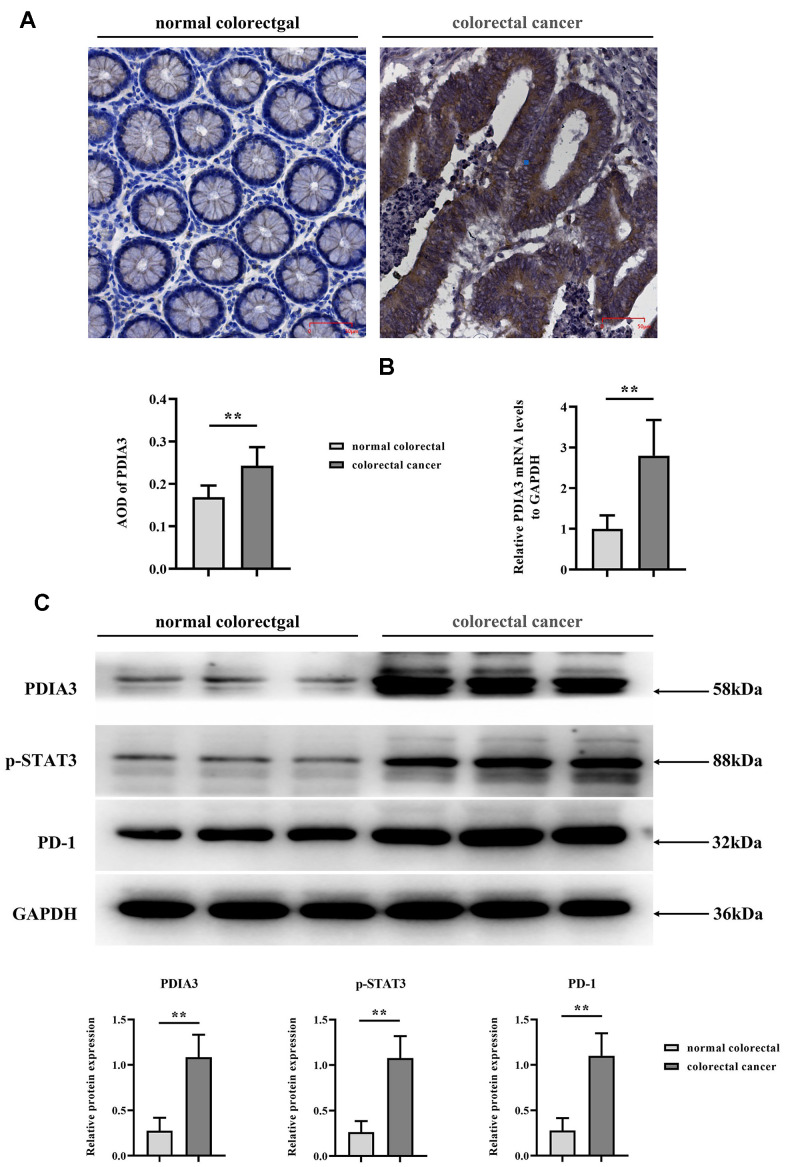
**Elevated levels of PDIA3, phosphorylated STAT3 (p-STAT3), and PD-1 are present in CRC tissues.** (**A**) Immunohistochemical assessment of PDIA3 protein in CRC and adjacent normative tissue. Statistical evaluation via T-test* *P < 0.01 versus normative control (n=96 pairs). (**B**) RT-qPCR quantification of PDIA3 mRNA in CRC and normative tissue (n=96 pairs). Statistical evaluation via T-test,* *P < 0.01 versus normative control. (**C**) Total protein isolation from tumor specimens with RIPA buffer, subsequently quantified via the BCA assay. Relative protein expression levels of PDIA3, p-STAT3, and PD-1 were determined after densitometric analysis from three independent samples. Statistical evaluation via T-test, * *P < 0.01 versus normative control.

Through the application of immunohistochemical assays (Figure 2A) and RT-qPCR (Figure 2B), we quantified PDIA3 levels within human colonic neoplasms versus normal colonic tissue (n=96 pairs). Results disclosed that colorectal carcinomas exhibited significantly increased PDIA3 expression. Additionally, Western Blot analysis delineated the expression differentials of PDIA3, phosphorylated STAT3, and PD-1 between healthy and cancerous colonic tissues (Figure 2C).

### The facilitative influence of PDIA3 on colorectal cancer progression

**Figure 3:**
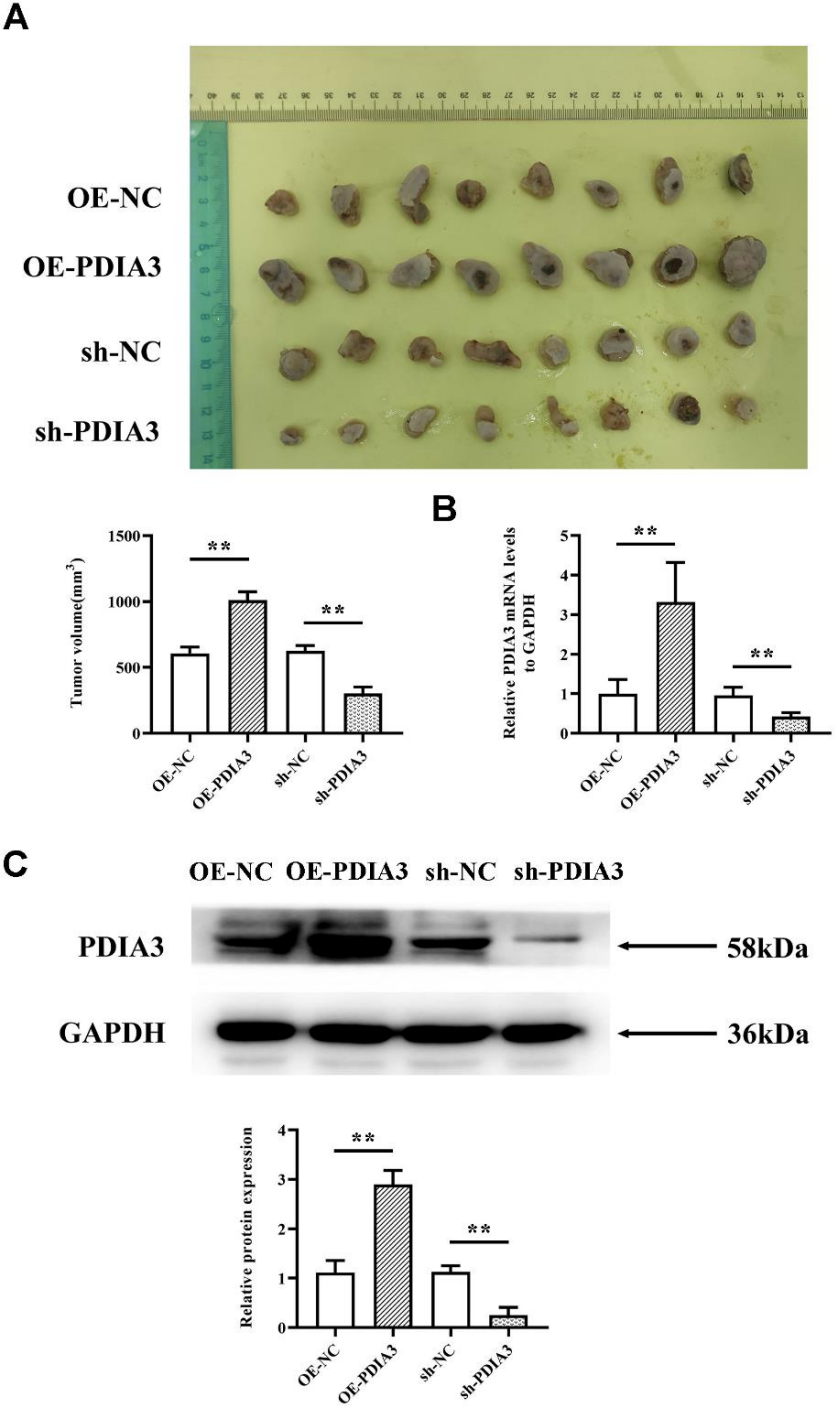
***In vivo* experiments demonstrate that PDIA3 significantly contributes to oncogenesis.** (**A**) SW480 cells, genetically modified to either overexpress or suppress PDIA3, were subcutaneously injected into nude mice to create CRC tumor models. Statistical evaluation via T-test,* *P < 0.01 versus normative control. (**B**) RT-qPCR detection of PDIA3 mRNA in CRC tumors. Statistical evaluation via T-test,* *P < 0.01 versus normative control. (**C**) Western blot detection of PDIA3 protein in CRC tumors. Statistical evaluation via T-test,* *P < 0.01 versus normative control.

Acknowledging PDIA3’s heightened expression in neoplastic conditions, we explored whether these observations are echoed in the genetically modified nude mouse model’s response to colorectal cancer. By implementing a subcutaneous xenograft model in BALB/c mice, we discerned that the sh-PDIA3 contingent demonstrated a notable tumor-suppressive effect when contrasted with the control cohort. Direct comparison between groups revealed significant tumoral amplification in PDIA3-overexpressing subjects and a substantial reduction in the sh-PDIA3 set, relative to controls (Figure 3A). This trend further extended to RNA and protein levels, corroborating PDIA3’s role in fostering colorectal carcinogenesis (Figure 3B, 3C).

### The role of PDIA3 protein within the STAT3/PD-1 signaling nexus

**Figure 4:**
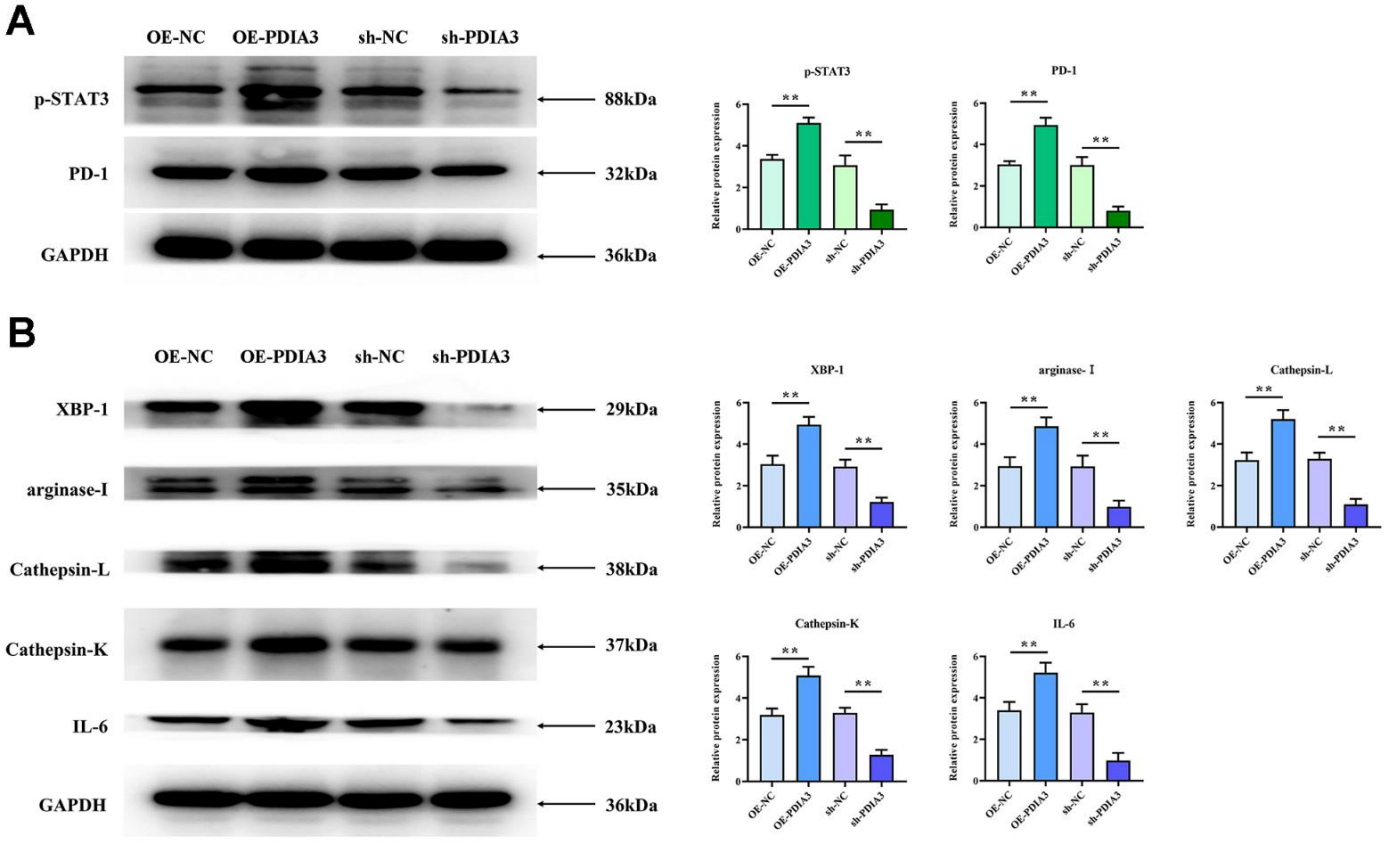
**PDIA3 facilitates M2 polarization of tumor-associated macrophages and augments tissue protease secretion through modulation of STAT3/PD-1 signaling.** (**A**) Comparison of p-STAT3 and PD-1 protein levels in cells with PDIA3 overexpression versus or-NC control, and PDIA3 knockdown versus sh-NC control, as determined by western blot (n=3 per group). Statistical evaluation via Ttest,* *P < 0.01 versus corresponding control. (**B**) Protein expression of XBP-1, arginase-1, cathepsin-L, cathepsin-K, and IL-6 in PDIA3-altered THP-1 cells, assessed by western blot (n=3 per group). Statistical evaluation via T-test,* *P < 0.01 versus corresponding control.

Subsequent *in vivo* experimentation authenticated PDIA3’s modulation of tumorigenicity in SW480. Probing the underpinnings of this phenomenon, we postulate a direct effect of PDIA3 on the STAT3 pathway, paralleling evidence extant in scholarly discourse and empirical observations. PDIA3, recognized as a protein disulfide isomerase, is implicated in the folding and corrective mechanisms of intracellular proteins. Phosphorylated STAT3 translocates to the nucleus, engaging specific gene promoters to govern gene expression and modulate macrophage polarization. Conversely, PD-1 operates as a suppressive immune checkpoint, its heightened expression fostering immune tolerance and blunting antitumor immunity. Western Blot evaluations demonstrated that the relative protein expressions of PDIA3, phosphorylated STAT3, and PD-1 were significantly accentuated in colorectal cancer tissues compared to their normal counterparts (Figure 4A), suggesting that PDIA3 is instrumental in regulating the STAT3/PD-1 signaling pathway.

### PDIA3 augments M2 polarization in tumor-associated macrophages and augments secretion of tissue proteases via modulation of the STAT3/PD-1 axis

**Figure 5:**
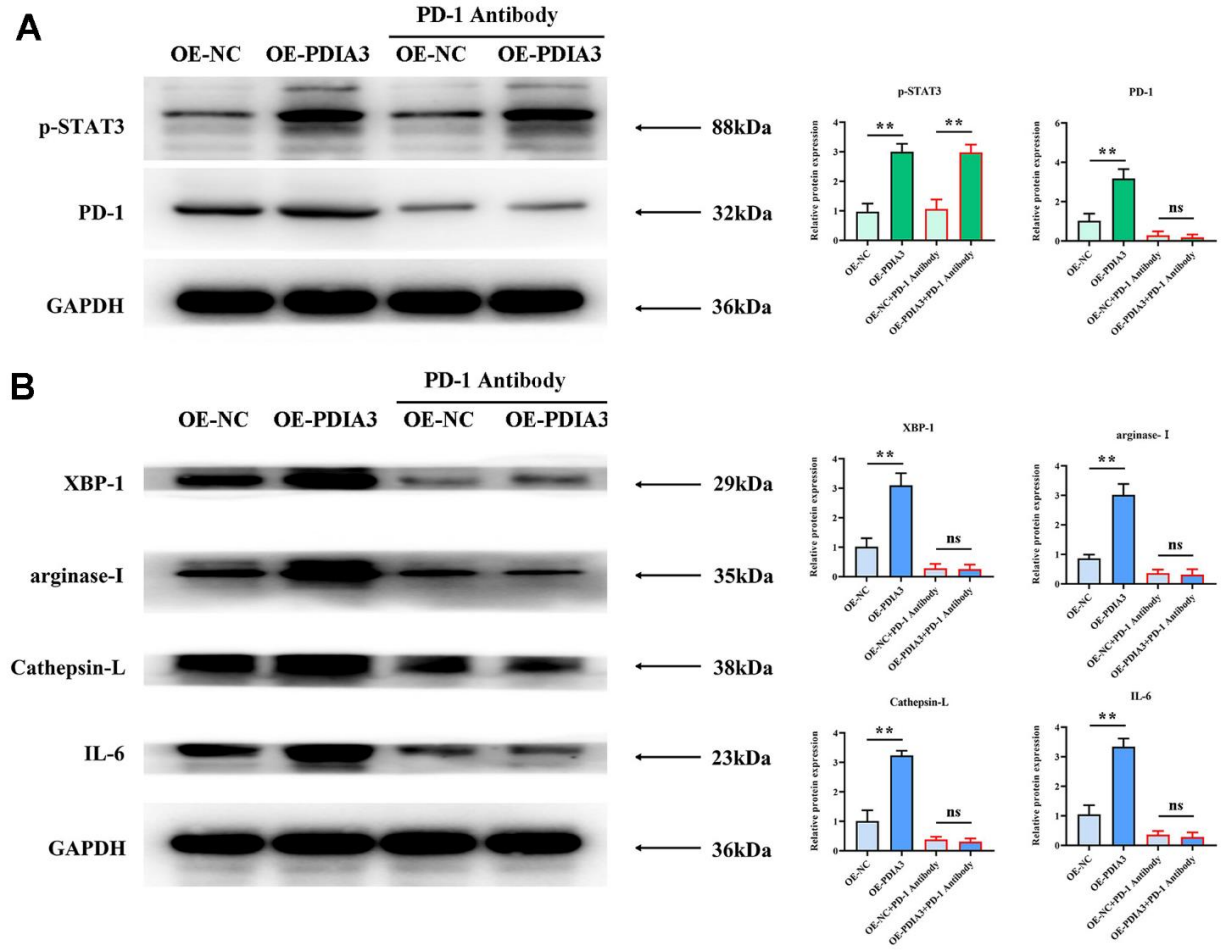
**Application of PD-1 antibody to substantiate PDIA3’s role in endorsing M2 polarization of tumor-associated macrophages and prompting tissue protease secretion, mediated by STAT3/PD-1 signaling modulation.** (**A**) Protein levels of pSTAT3 and PD-1 in cells with PDIA3 overexpression versus or-NC control, and PD-1 inhibitor versus PD-1 NC treatment assessed by western blot (n=3 per group). Statistical evaluation via T-test,* *P < 0.01 versus corresponding control. (**B**) Levels of XBP-1, arginase-1, cathepsin-L, cathepsin-K, and IL-6 proteins in THP-1 cells under differing conditions of PDIA3 expression and PD-1 inhibition, determined via western blot (n=3 per group). Statistical evaluation via T-test,* *P < 0.01 versus corresponding control.

Substantial evidence has emerged linking PDIA3 to the promotion of M2-polarization in tumor-associated macrophages and the enhancement of tissue protease secretion, mediated by the STAT3/PD-1 signaling cascade. Correlations between PD-1 and XBP-1 suggest that activation of the PD-1 pathway may modulate XBP-1 expression, influencing both macrophage polarization and protease secretion. Western blot analyses reveal markedly elevated expression levels of phosphorylated STAT3 (p-STAT3), PD-1, XBP-1, arginase-1, cathepsin-L, cathepsin-K, and interleukin-6 (IL-6) in SW480 cells with PDIA3 overexpression (OE-PDIA3 group) and in THP-1 cells, in stark contrast to the control group (PDIA3-NC group). Conversely, suppression of PDIA3 expression using shRNA (PDIA3-shRNA group) led to a significant reduction in these proteins’ expression levels in SW480 and THP-1 cells (Figure 4B). The application of a PD-1 monoclonal antibody abrogated the disparities in protein expression observed between the OE-PDIA3 and PDIA3-NC groups. These findings reinforce the premise that PDIA3 is instrumental in fostering M2 polarization in tumor-associated macrophages and in the augmented secretion of tissue proteases, operations it conducts through the strategic regulation of the STAT3/PD-1 signaling pathway (Figure 5A, 5B).

### PDIA3 catalyses the motility of colorectal cancer cells by governing the STAT3/PD-1 pathway, alongside modulating macrophage M2 polarization and protease secretion

**Figure 6:**
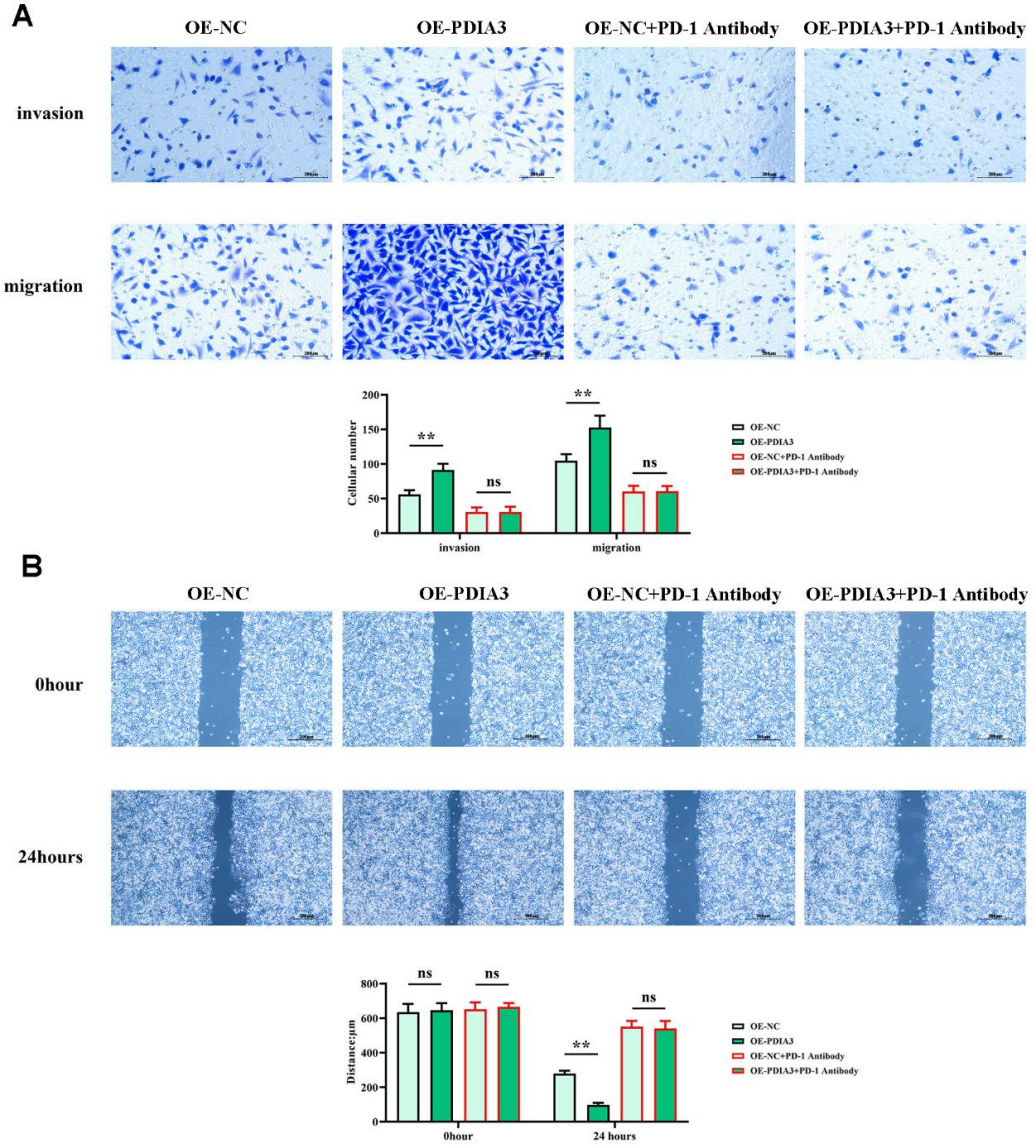
**PDIA3 enhances the migratory capacity of CRC cells through the regulation of STAT3/PD-1 signaling, along with influencing tumor-associated macrophage M2 polarization and tissue protease secretion.** (**A**) Transwell migration and invasion assay results for SW480 cells, with comparative analysis between PDIA3 overexpression and OE-NC control, and PD-1 inhibitor and PD-1 NC treatments (n=3 per group, magnification 200x). Statistical evaluation via T-test,* *P < 0.01 versus corresponding control. (**B**) Wound healing assay results depicting cellular migration over 24 hours between SW480 cells with PDIA3 overexpression versus or-NC control, and PD-1 inhibitor versus PD-1 NC treatments (n=3 per group, magnification 200x). Statistical evaluation via T-test,* *P < 0.01 versus corresponding control.

Empirical evidence from *in vitro* assessments has unveiled PDIA3’s dual role in enhancing the migratory prowess of colorectal cancer cells. While reconciling with *in vivo* corroboration, this underscores its cardinal influence. Scrutiny of cell migration through wound-healing assays (Figure 6B) uncovered a pronounced constriction of inter-scratch gaps in the PDIA3 overexpression cohort relative to controls at the 24-hour mark. This differential, however, was rendered inconsequential by the intervention of a PD-1 monoclonal antibody. In corroborating Transwell assays (Figure 6A), a stark increase in cellular transmigration was observed in the OE-PDIA3 samples vis-à-vis the PDIA3-NC control. Here too, PD-1 antibody inclusion neutralized the divergence. These results collectively infer that PDIA3 is a pivotal agent in propelling the migratory capabilities of colorectal cancer cells through its modulatory effects on the STAT3/PD-1 signaling nexus as well as macrophage M2 polarization and tissue protease secretion.

### PDIA3 invigorates the proliferative dynamics of colorectal cancer cells via the STAT3/PD-1 network, coupled with modulating macrophage M2 polarization and protease secretion

**Figure 7:**
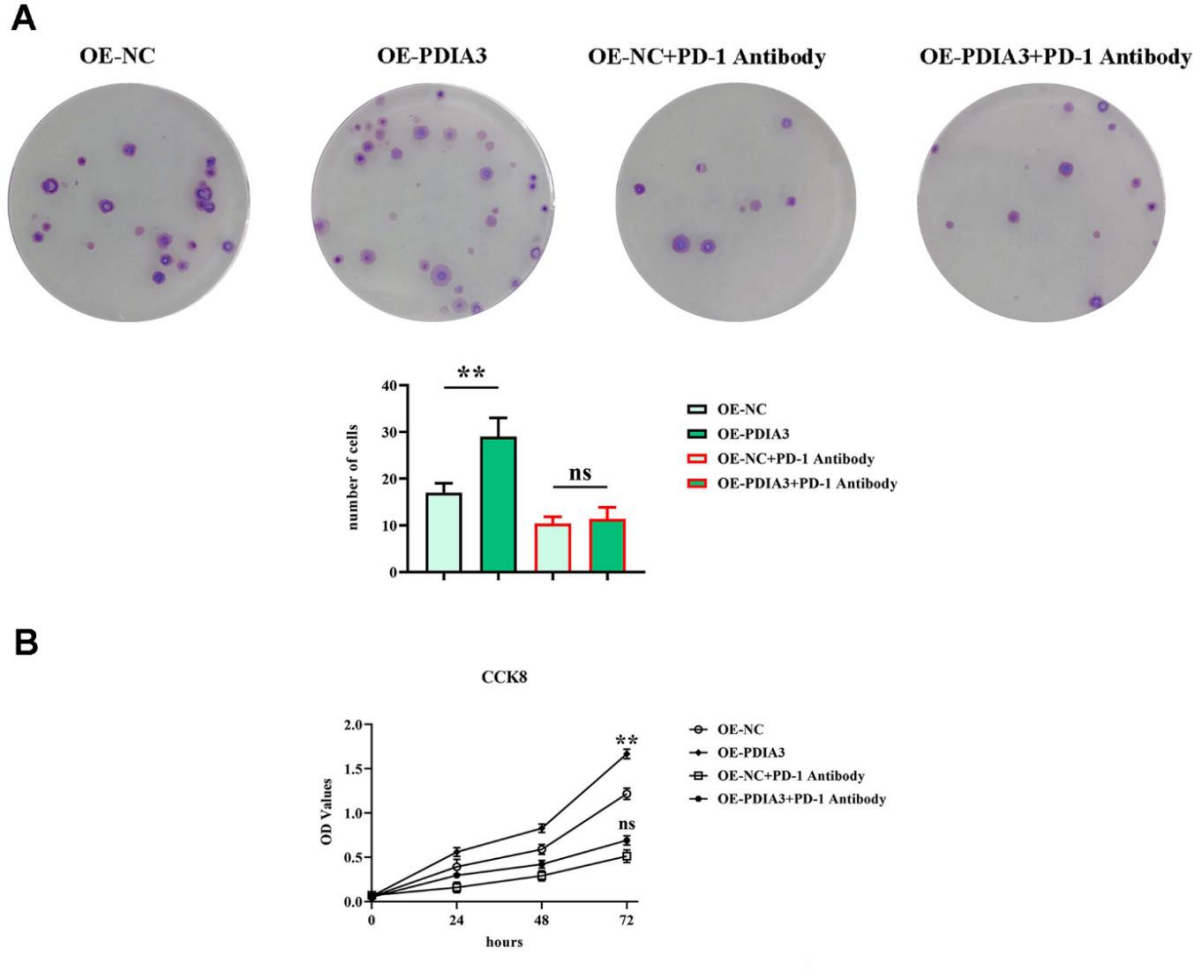
**PDIA3 promotes CRC cellular proliferation by modulating STAT3/PD-1 signaling pathway, facilitating tumor associated macrophage M2 polarization, and increasing tissue protease secretion.** (**A**) Colony formation assay highlighting the differences in colony numbers of SW480 cells between PDIA3 overexpression versus or-NC control, and PD-1 inhibitor versus PD-1 NC treatments, observed after 14 days (n=3 per group). Statistical evaluation via T-test,* *P < 0.01 versus corresponding control. (**B**) CCK8 assay provides a quantitative comparison of cell proliferation rates at 24, 48, and 96 hours, indicated by absorbance measurements at 450 nm for SW480 cells with PDIA3 overexpression versus or-NC control, and PD-1 inhibitor versus PD-1 NC treatments (n=3 per group). Statistical evaluation via T-test,* *P < 0.01 versus corresponding control.

Investigative methodologies involving colony formation and CCK-8 assays (Figure 7A, 7B) were employed to discern the impact of PDIA3 on the proliferative competency of colorectal cancer cells. Although prior revelations have expounded its role in migration and invasion, the question of its influence on cell proliferation merited elucidation. Outcomes from clonal assays (Figure 7A) manifested a discernible amplification in colony counts and optical density (OD) readings within the OE-PDIA3 cohort, diverging noticeably from the PDIA3-NC group. The administration of PD-1 monoclonal antibodies effectively nullified the observable differences in colony proliferation and OD measurements across the experimental divides. Thus, it is evident that PDIA3 stimulates the proliferative capacity of colorectal cancer cells by masterminding the STAT3/PD-1 signaling paradigm, in tandem with refining macrophage M2 polarization and the secretion of tissue-specific proteases.

### The role mechanism of PDIA3 in the tumor microenvironment - schematic diagram

**Figure 8:**
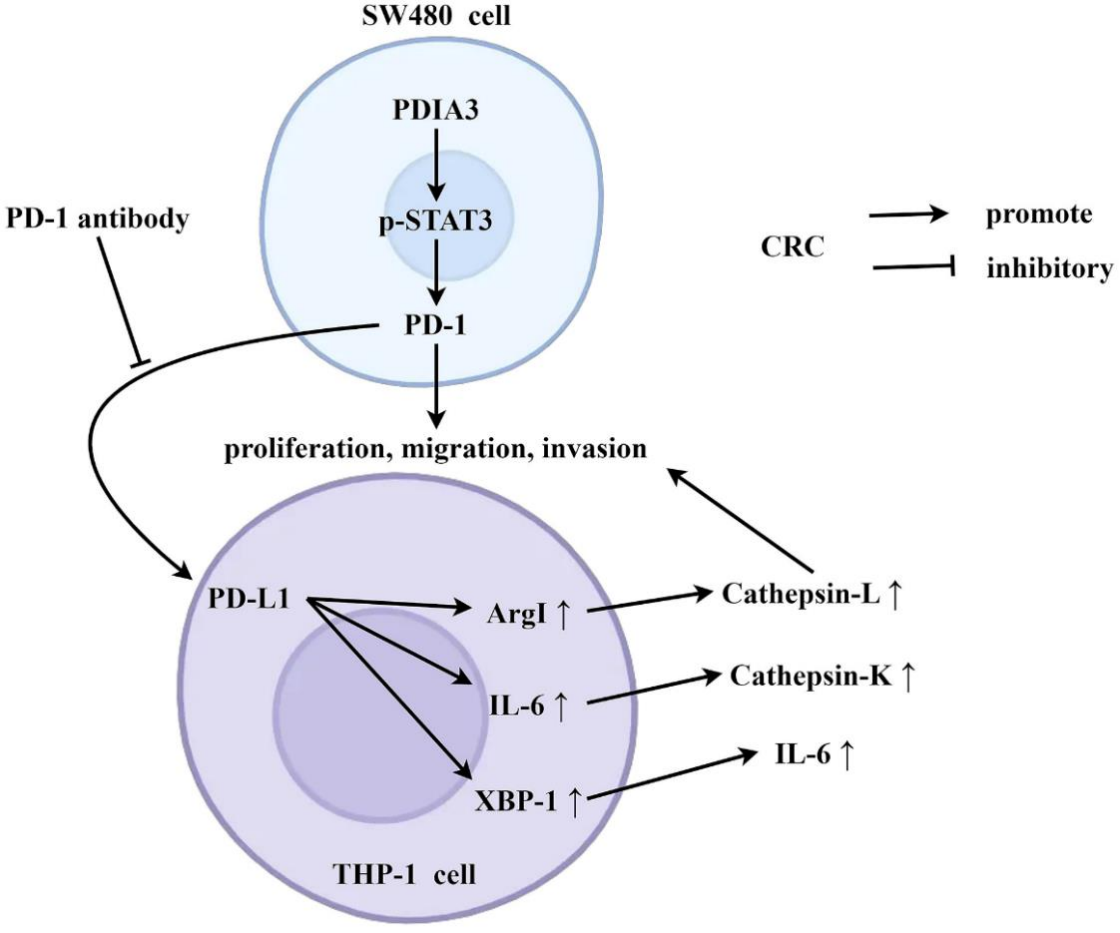
**PDIA3 is pivotal in accentuating colorectal cancer proliferation and metastasis, orchestrated through strategic regulation of the STAT3/PD-1 signaling axis and the concurrent promotion of tumor-associated macrophage M2 polarization.** Under the condition of co-culture of tumor-associated macrophages and SW480 cells, PDIA3 can promote the expression of PD-1 by enhancing the phosphorylation of STAT3, and PD-1 is then secreted extracellularly. PD-1 acts on the PD-L1 receptor of SW480 cells, thereby promoting the expression of arg1, IL-6, and XBP-1, and inhibiting the secretion of cathepsin L, cathepsin K, and IL-6. This further promotes the migration, invasion, and proliferation abilities of SW480 cells.

Under the condition of co-culture of tumor-associated macrophages and SW480 cells, PDIA3 can promote the expression of PD-1 by enhancing the phosphorylation of STAT3, and PD-1 is then secreted extracellularly. PD-1 acts on the PD-L1 receptor of SW480 cells, thereby promoting the expression of arg1, IL-6, and XBP-1, and inhibiting the secretion of cathepsin L, cathepsin K, and IL-6. This further promotes the migration, invasion, and proliferation abilities of SW480 cells (Figure 8).

## DISCUSSION

Carcinoma looms as a predominant arbiter of mortality amongst both genders on a global scale. Specifically, colorectal carcinoma (CRC) constitutes a notable ten percent of total newly diagnosed carcinoma occurrences, heralding an excess of four percent lifetime probability of contracting the malady [22–25]. Contemporary zeniths in research, namely cancer immunotherapy, which includes checkpoint inhibitors and adoptive cellular therapy, strategize to recalibrate the immune system for the identification and eradication of neoplastic cells. A luminary target within this therapeutic domain for addressing prostate carcinoma is the PDIA3 protein [26, 27]. The realms of biotechnology and microbial studies ceaselessly advance in devising innovative curatives for an array of carcinomas, CRC included, proffering a glimmer of solace for those besieged by this scourge. PDIA3 shines as an emergent biomarker in endometrial carcinoma [28] and is furthermore cataloged as an immune checkpoint hallmark within gliomas [29]. Its integration in the assessment, diagnostic process, and stratification of colorectal carcinoma is posited as a pivotal benchmark for precocious detection [30].

Delving into the TIMER2.0 compendium, an intimate linkage emerges between PDIA3 and the genesis and progression of sundry tumors, with its heightened expression in CRC tissues. With the employment of the “corrplot” apparatus within the R programming milieu, we delineated a robust positive interconnection between PDIA3 with STAT3, in tandem with a congruence with CD274 expression. The confluence of PDIA3 and STAT3 is substantiated; PDIA3’s ability to engage directly with genomic strands in the nucleus, or potentiate the genomic strand nexus of the STAT3 complex, potentiates said transcription factor’s translocation from the nucleus, thereby influencing gene expression governed by STAT3. The versatile engagements of PDIA3 in cellular communiques, including interplays with STAT3 that impart influence upon mitochondrial respiration, are mirrored by inquiries revealing augmented mitochondrial functionality in PDIA3-deficient cells—a phenomenon later abated by a STAT3 antagonist. Investigations purport a tandem ascent of PDIA3 and PD-L1 levels in CRC evolution. An escalation of PDIA3 expression may provoke a commensurate elevation of both PD-1 and potentially PD-L1. Considering that PD-L1, sporting the guise of immune cell membranes, coalesces with PD-1 to curtail the activation and cytotoxic prowess of the same towards neoplastic cells, a surge in PDIA3 could champion the veiling of tumor entities from immune surveillance. Notwithstanding these insights, a call for painstaking exploration endures to decode the intricate relationship between PDIA3 and CD274, and how this nexus might be transmuted into therapeutically viable inroads in oncological treatments. Additionally, analyses sourced from the TIMER2.0 archive unveiled a tangible congruence between CD274 expression and the presence of monocytes/macrophages. The genesis of a subdermal neoplasm-bearing murine model in Balb/c mice permitted us to ascertain that the cohort subjected to PDIA3 suppression manifested a pronouncedly antagonistic effect on the tumor compared to the control ensemble.

The administration of the STAT3 inhibitor napabucasin efficaciously thwarted neoplastic proliferation while concurrently eliciting the upregulation of calreticulin and Protein Disulfide Isomerase Family A Member 3 (PDIA3, also known as ERp57). However, this treatment resulted in the diminution of CD47 and GLUT1 expression. Moreover, the density of dendritic cells (DCs) and macrophages infiltrating the tumor matrix saw a marked increase, accompanied by heightened expression of costimulatory molecules. A notable accrual of CD4+ and CD8+ T-lymphocytes was observed within the neoplastic tissues, wherein CD8+ T-cells exhibited reduced expression of regulatory checkpoint molecules, such as the lymphocyte activation gene-3 protein (LAG-3) and the programmed cell death protein 1 (PD-1) [31]. Notably, PDIA3 hindered mitochondrial energetics via the inhibition of STAT3 serine 727 phosphorylation [32]. Furthermore, PDIA3 has been implicated in the progression of hepatocellular carcinoma through the modulation of the STAT3 signaling cascade [18].

A comparative analysis of neoplastic data between cohorts revealed a significant augmentation in tumor volume within the PDIA3 overexpression group, contrasted with a considerable reduction in the sh-PDIA3 cohort as compared to the normoexpressed controls. The role of PDIA3 as an impetus in colorectal carcinogenesis is thus exemplified. Corroborating evidence was obtained through focused examinations utilizing specimens from 96 patients afflicted with colorectal carcinoma. Immunohistochemical evaluations disclosed conspicuously elevated levels of PDIA3 in colorectal neoplasms in comparison to healthy colonic tissues—a finding substantiated by Western blot analyses, which indicated pronounced PDIA3 expression in tumor samples. Concomitantly, the expression profiles of phosphorylated STAT3 and PD-1 were observed to surge parallel to PDIA3 upregulation. Prior research, employing proteomics technology to detect PDIA3 in colorectal cancer patients, aligns with our observed expression modifications within the tumor tissue. Hence, we postulate that PDIA3 plays an integral role in the pathogenesis and progression of colorectal cancer.

Tumor-associated macrophages (TAMs) are implicated as pivotal players in the pathophysiology of colorectal carcinoma [33]. Functioning as crucial constituents of the immune architecture, macrophages orchestrate inflammation, tissue restitution, immune surveillance, and an array of vital processes. TAMs manifest predominantly in two phenotypical expressions: the M1 type, associated with anti-tumorigenic activities, and the M2 type, conducive to tumorigenesis [34]. In the tumor milieu, macrophages may undergo metamorphosis in their functionality and phenotype, segueing into TAMs typified by M2 polarization, known for facilitative roles in neoplastic proliferation and dissemination [35]. Amidst substantial PDIA3 expression in colorectal cancer tissues, we manipulated PDIA3 protein levels within TAMs to decrypt the protein’s function in colorectal neoplasia [32]. Intriguingly, M2 macrophages have been identified as facilitators that aid neoplastic cells in eluding immunological defenses across various malignancies. This delineates a prospective link between PDIA3 functions and M2 macrophage operation. PDIA3, with its intricate influence on protein structuring and signal mediation, may sway the operational integrity of M2 macrophages, potentially casting effects upon the immune responsivity and consequently influencing conditions such as neoplastic development and inflammation-related disorders.

Precise elucidation of the mechanisms by which PDIA3, alongside STAT3 and the PD-1/PD-L1 axis, influences therapeutic denouements in colorectal cancer remains quintessential. Expeditions in research voraciously dissect avenues to surmount treatment resistance and to forge novel, ostensibly more efficacious, and benign therapeutic regimens that singularly target these proteins and their associated pathways. For example, amalgamating inhibitors of PD-1/PD-L1 with alternative therapeutic stratagems could potentially dispel resistance and amplify therapeutic success in individuals with colorectal cancer. It is the fervent hope that, with advancing scientific inquiry, strategies will be refined to mitigate the unwelcome effects of drugs and dismantle the resistance mechanisms that undermine the potency of extant therapy for colorectal malignancy.

Evolving research delineates that the heightened expression of PDIA3—Protein Disulfide Isomerase Family A Member 3—nurtures the synthesis of phosphorylated Signal Transducer and Activator of Transcription 3 (p-STAT3), which, in turn, occasions the upsurge of Programmed Death 1 (PD-1). Upon being effused into the extracellular milieu, PD-1 appears to exert its function by engaging with macrophages, influencing their differentiation trajectory. This chain of events orchestrates the transmutation of macrophages from an undifferentiated M0 state to an M2-type macrophage. This metamorphosis is characterized by the secretion of biomarkers such as Arginase 1 (Arg1) and Interleukin-6 (IL-6), suggesting a predilection toward an anti-inflammatory and tissue remodeling ethos rather than a pro-inflammatory one. Additionally, the upswing of matrix metalloproteinases intimates an augmented capacity for tissue restructuring. Such development of an M2 macrophage domain is less propitious in mounting an immune onslaught against colorectal cancer cells, attenuating the cytotoxic reprisal that typically reins in cancer evolution. This scenario portends a weakened immune stronghold, permitting malign cells to shroud themselves, potentially spurring tumor advancement and metastasis. This underscores the critical role of the immune system, with macrophages as a central focus, within the purview of cancer immunology and underscores the potential merit in effectuating therapeutic strategies targeted at the PDIA3/p-STAT3/PD-1 nexus as a means to confront colorectal cancer and plausibly other malignancies where similar molecular pathways prevail.

Immunoblot analyses illuminated that, in the PDIA3-deficient control cohort, there was a notable augmentation in the relative expression levels of proteins such as phosphorylated-STAT3, PD-1, XBP-1, arginase-1, cathepsin-L, cathepsin-K, and IL-6 within the PDIA3-overexpressing (OE-PDIA3) factions of SW480 and THP-1 cells. In stark contrast, these respective protein expression levels demonstrated a pronounced decrement in SW480 and THP-1 cells treated with PDIA3-targeted shRNA. Following the introduction of a PD-1 monoclonal antibody, the expression levels of phosphorylated-STAT3 in the OE-PDIA3 assemblage remained consistent, while the disparities in expression of PD-1, XBP-1, arginase-1, cathepsin-L, cathepsin-K, and IL-6 vis-à-vis the PDIA3 control group were effectively abrogated. Investigations have revealed an intimate biochemical liaison between PDIA3 and STAT3, whereby an increased expression of PDIA3 escalates STAT3 phosphorylation and functional prowess, thereby potentiating the activation of the STAT3 signaling cascade and its associated transcriptional governance over a plethora of cellular processes encompassing vitality, proliferation, and immunomodulation. Such revelation posits that PDIA3 may champion the M2 polarization of tumorigenic macrophages and the excretion of tissue proteolytic enzymes through finesse in the STAT3/PD-1 signal transduction pathway.

Delving into the cellular mechanics of wound healing and translocation, our empirical scrutiny has discerned that PDIA3 considerably escalates the migratory tendency of malignant colorectal entities. This facilitation takes a route through the delicate orchestration of the STAT3/PD-1 signaling confluence, contributing to a heightened state of M2 polarization in tumor-associated macrophages as well as a surge in the liberation of tissue proteases. Concurrently, evaluations relating to clonal propagation have laid bare a significant upsurge in clonal conglomeration within collectives overexpressing PDIA3 when appraised against their PDIA3-devoid counterparts, a surge that was nullified post hoc with the incorporation of a PD-1monoclonal antibody. Aligning with these insights, cell viability examinations, epitomized by the CCK-8 assay, have divulged a noteworthy ascent in optical density measurements at the 72-hour mark after cultivation within PDIA3-overexpressing contingents—an elevation rendered inconsequential upon the advent of the PD-1 monoclonal antibody. These revelations intimate that PDIA3 may contribute to the enhanced proliferation of colorectal cancer cells through its masterful orchestration of the STAT3/PD-1 signaling axis, thereby engendering an enriched environment for M2 macrophage polarization and protease secretion. The entanglement of PDIA3 with other immune checkpoints rests as an absorbing domain for future inquiry, poised to unravel the spectrum of immune evasion stratagems at play within the realm of colorectal neoplasia. Delving deeper into the role of PDIA3 in resistance to immunotherapy surfaces as a pressing investigative trajectory. Integration of strategies aimed at modulating PDIA3 within the perimeters of clinical experimentation may well forge the foundation stone for the evolution of tailored therapeutic tact. Forthcoming scholarly pursuits might well appraise the prognostic precision of PDIA3’s expression levels, thereby assisting in the finesse of patient stratification and the monitoring of therapeutic impact. PD-1, stationed downstream of PDIA3, is poised to influence subsequent proliferation, migration, and invasion which, when challenged by PD-1 antibodies, is markedly subdued, consequently attenuating the capabilities of proliferation, migration, and invasion.

Comprehensive bioinformatics analysis revealed a significant increase in PDIA3 expression in colorectal cancer, associated with STAT3, CD274, and markers of monocytic/macrophage lineage. Utilizing the TIMER2.0 database, overexpression of PDIA3 in colorectal tumors was initially identified, and further confirmed through GEO database analysis, establishing a strong correlation between PDIA3, STAT3, and CD274. Additionally, immunohistochemical and molecular techniques substantiated the elevated levels of PDIA3 in colorectal carcinoma tissue compared to normal colon tissue, highlighting the prevalence of this protein in carcinogenic samples. Investigations into the role of PDIA3 in colorectal cancer progression revealed its significant influence on disease development, evidenced by a xenograft model in nude mice showing a reduction in tumor growth with PDIA3 suppression. Furthermore, *in vivo* studies confirmed PDIA3’s ability to modulate the STAT3 pathway, affecting macrophage polarization and contributing to an immunosuppressive environment via PD-1 elevation, as demonstrated through comparative protein expression analyses. Intriguingly, PDIA3 was found to promote an M2 macrophage phenotype in tumor-associated macrophages and increase tissue protease secretion through the STAT3/PD-1 axis. The alteration of key proteins, including PD-1 and XBP-1, in response to PDIA3 overexpression or suppression, corroborated the pivotal role of PDIA3 in tumor-associated macrophage polarization and protease production, as confirmed by Western blotting and the effect of PD-1 antibodies in these processes. Moreover, PDIA3 was demonstrated to catalyze colorectal cancer cell motility by governing the STAT3/PD-1 pathway, while simultaneously modulating M2 macrophage polarization and protease secretion. Wound-healing and Transwell assays provided evidence of PDIA3’s impact on cancer cell migration, with the introduction of PD-1 antibodies reversing the enhanced migratory effects observed in PDIA3-overexpressing cells. Finally, PDIA3 was implicated in the proliferative dynamics of colorectal cancer cells through the STAT3/PD-1 network. Colony formation and cellular viability assays delineated the proliferative boost imparted by PDIA3, with PD-1 antibody treatment effectively mitigating these effects, thereby underscoring PDIA3’s crucial role in regulating both cell proliferation and the immune signaling interface within the colorectal cancer milieu.

To encapsulate, our inquiry delved into the expression of PDIA3 in the tissues afflicted by colorectal carcinoma. Cellular experimentation underpinned the proposition that PDIA3 impels the M2 polarization of tumor-aligned macrophages, alongside fostering proliferation and the potential for metastasis in colorectal cancer, all by the delicate modulation of the STAT3/PD-1 signaling continuum. These insights offer a novel therapeutic target for combating colorectal cancer, with intervention strategies targeting PDIA3 shimmering with the potential to ameliorate colorectal cancer prognosis.

## MATERIALS AND METHODS

### Patient sample collection

A total of 96 colorectal cancer (CRC) patients were recruited from the Department of General Surgery, Affiliated First Hospital of Hebei North University. Informed consent was obtained from all participants, and the study was approved by the Ethics Committee of Affiliated First Hospital of Hebei North University (ApprovalNo.20220102003). None of the patients had received radiotherapy or chemotherapy before surgery. All samples were immediately frozen in liquid nitrogen after surgery and then later stored at -80° C for further use.

### Bioinformatics analysis

Based on UCSC Xena (http://xena.ucsc.edu/) Download transcriptome data (log2 (counts+1) values) and clinical data from 41 normal colon cancer tissues and 471 tumor tissues using the Cancer Genome Atlas (TCGA) database for subsequent analysis. Download independent datasets from the GEO database for external validation, GSE20916 includes 44 normal colon samples, 55 adenomas, and 36 adenocarcinoma tissue samples to verify the differential expression of the PDIA3 gene. Comparisons of differential expression of PDIA3 in pan-cancer were performed using the TIMER2.0 database. The expression matrices of PDIA3 in normal tissues and tumor tissues were obtained from the GEO (GSE20916) database. The “corrplot” package in R language calculates the correlation and P-value of PDIA3 and STAT3, as well as PDIA3 and CD274, based on the Pearson correlation algorithm, and draws a scatter plot. The TIMER2.0 database is used to calculate the correlation between CD274 and monocytes/macrophages. Obtain overall survival and disease-free survival rates of PDIA3 in colorectal cancer from the GEPIA database.

### Immunohistochemical analysis

Select 96 pairs of CRC patient tissues and normal intestinal tissues, take an appropriate amount of size, and place it in a tissue embedding box. Fix it with 10% neutral buffered formalin solution, embed the fixed tissues with paraffin, and use a slicer to cut the paraffin tissue into 4 sections μM tissue slices were dewaxed with xylene and ethanol, and antigen repair was performed on the tissue using sodium citrate antigen repair solution. Afterward, endogenous peroxidase blockers were dripped onto the surface of the tissue and then blocked with 5% goat serum. Slices were incubated overnight with ERp57 Rabbit mAb at 4° C, followed by incubation at room temperature with enzyme-linked goat anti-rabbit IgG polymer for 20 minutes. Add the prepared DAB colorimetric solution onto the tissue and cover the entire tissue. Stop staining when the reaction turns brownish-yellow. Hematoxylin re-stained cells. Hydrochloric acid alcohol differentiation, anti-blue. Gradient alcohol dehydration, transparent xylene. Neutral gum seal. Use Leica microscopy imaging system to capture images of tissue slices. Dyeing evaluation criteria: Grey density analysis method. Immunohistochemical images were identified and analyzed using ImageJ Fiji (National Institutes of Health, NIH) software to obtain AOD values. Then, significance analysis was performed using GraphPad Prism9.0 statistical software.

### Cell culture and grouping

Human colorectal cancer cell line SW480 and human monocytic cell line THP-1 were obtained from Wuhan Pusen Bioscience Ltd., Hubei, China. The cells were cultured in the complete growth medium, DMEM (PM150210), supplemented with 10% fetal bovine serum (164210-50) and 1% penicillin-streptomycin (PB180120), and maintained in a humidified incubator at 37° C. When the SW480 cells reached approximately 80% confluency, the culture medium was replaced with RPMI1640 medium without fetal bovine serum. According to the instructions of the Lipofectamine TM2000 transfection reagent kit, sh-NC, sh-PDIA3, and OE-PDIA3 each 0.5ug was transfected into SW480 cells. After 6 hours of transfection, the medium was replaced with a fresh RPMI1640 medium containing 10% fetal bovine serum, and the cells were further incubated in a six-well plate for 48 hours. Subsequently, the SW480 cells were placed in transwell chambers for indirect co-culture with THP-1 cells.

### Animal study and establishment of the CRC mouse model

Male BALB/c mice (6-8 weeks old) were purchased from Spafas (Beijing, China) Experimental Animal Technology Co., Ltd. All *in vivo* manipulations on the mice were carried out by the requirements of Hebei North University (Approval number: HBNU20220107000016). Mice were randomly divided into four groups of 8 mice in each group to establish a mouse subcutaneous tumor model. Groups of cells (2x10^6^ cells/mouse) are injected subcutaneously into the mouse. Tumor volume is calculated after 10 days. The animals were housed under specific pathogen-free conditions in an environment with regulated temperature (25±1°C) and humidity (40-70%) and exposure to a constant 12-h light/dark cycle in the animal facility at Affiliated First Hospital of Hebei North University.

### RT-qPCR

The RNeasy Midi Kit was used for RNA extraction according to the manufacturer’s instructions. The entire isolated RNA was dissolved in 300μl RNase-free water. RNA purity and concentration were evaluated by optical density measurement by applying a Nano-Drop spectrophotometer (Bio-Tek, USA) and 3μg of RNA in 3 aliquots underwent reverse transcription. real-time RT-qPCR assays were constructed using the SYBR Green PCR Kit in the CFX96 real-time PCR detection system (BioRad, USA). Assessment of gene expression markers was made in 3 separate vials. The primer for PDIA3, F: 5-AAGCAGCGGGTTAGTGGTC-3; R:5-TCGAAGTTGTCGTCCGTGAG-3.

### Western blot

Proteins from tissues and cells were lysed using RIPA lysis buffer containing proteinase and phosphatase inhibitors. Protein quantification was performed using the Pierce™ BCA Protein Assay Kit, following the manufacturer’s protocol. 10% SDS-PAGE separating gel and 5% SDS-PAGE stacking gel were prepared and placed in an electrophoresis tank filled with 1× running buffer. Proteins were loaded, and electrophoresis was conducted until the bromophenol blue indicator reached the bottom of the separating gel. The proteins from the 10% SDS-PAGE gel were transferred onto a PVDF membrane, which was then blocked with 5% skim milk at room temperature for 1 hour. Primary antibodies against PDIA3 (1:1000, Invitrogen), p-STAT3 (1:1000, Invitrogen), PD-1 (1:1000, Invitrogen), XBP-1 (1:1000, Invitrogen), arginase-1 (1:1000, Invitrogen), cathepsin-L (1:1000, Invitrogen), cathepsin-K (1:1000, Invitrogen), IL-6 (1:1000, Invitrogen) and GAPDH (1:1000, Invitrogen) were incubated with the PVDF membrane overnight at 4° C. After that, the membrane was incubated with secondary antibodies at room temperature for 90 minutes. The immune complexes were detected using an automated chemiluminescence imaging system.

### Wound healing experiment

Parallel lines were drawn on the floor of a 6-well plate using a marker. SW480 cells were seeded onto the plate and cultured until reaching a cell density of 1×10^5^ cells/ml. The next day, a scratch was made vertically across the cell monolayer using the tip of a sterile pipette, with a 4 mm gap between the scratch lines. After washing with PBS, the serum-free medium was added, and the plate was placed in a humidified incubator containing 95% O_2_ and 5% CO_2_ at 37° C. Images were taken at 0 hours and 24 hours. The width of the scratch was calculated using Image-Pro Plus software.

### Transwell assay

Transwell chambers with 24-well plates and an 8.0μm pore-size polycarbonate membrane were used. SW480 cells were seeded at a density of 1×10^5^ cells/well in 100μL serum-free medium in the upper chamber, while 600μL of complete growth medium was added to the lower chamber as a chemoattractant. After incubation at 37° C for 48 hours, residual cells on the upper surface of the membrane were removed using a cotton swab, and the migrated cells on the lower surface of the membrane were fixed with 4% paraformaldehyde and stained with 0.1% crystal violet solution. The cells were then imaged using an inverted fluorescence microscope with a filter.

### Colony formation assay

SW480 cells were collected, trypsinized, counted, and plated in a 37° C humidified incubator for approximately two weeks until visible colonies formed. The medium was then aspirated, and the cells were washed three times with PBS. The cells were fixed with methanol for 15 minutes, air-dried, and stained with crystal violet for 30 minutes. After air-drying, the cells were scanned and photographed, and the number of visible cell colonies was counted.

### CCK-8 assay

The CCK-8 assay kit was used to assess the effects of different treatments on cell proliferation and toxicity. After washing the cells, they were digested with trypsin and seeded into a 96-well plate at a density of 1000 cells per well. The plate was incubated in a humidified incubator for an appropriate period (24h, 48h, and 72h). After this, the plate was removed from the incubator, and 10μL of CCK-8 solution was added to each well. Then the plate was incubated for an additional 2 hours in the incubator, and the absorbance at 450nm was measured using a microplate reader.

### Statistical analysis

Data analysis and plotting were performed using GraphPad Prism 9.0 software. Normally distributed measurement data were presented as mean + SEM. The independent sample t-test was used for comparisons between two groups, and one-way analysis of variance (ANOVA) was used for comparisons between multiple groups. P-values less than 0.05 were considered statistically significant.

### Data availability

The data that support the findings of this study are available from the corresponding author upon reason able request.

## References

[1] Siegel RL, Wagle NS, Cercek A, Smith RA, Jemal A. Colorectal cancer statistics, 2023. CA Cancer J Clin. 2023; 73:233–54. 10.3322/caac.21772 36856579

[2] Gobert AP, Latour YL, Asim M, Barry DP, Allaman MM, Finley JL, Smith TM, McNamara KM, Singh K, Sierra JC, Delgado AG, Luis PB, Schneider C, et al. Protective Role of Spermidine in Colitis and Colon Carcinogenesis. Gastroenterology. 2022; 162:813–27.e8. 10.1053/j.gastro.2021.11.005 34767785 PMC8881368

[3] Sung H, Ferlay J, Siegel RL, Laversanne M, Soerjomataram I, Jemal A, Bray F. Global Cancer Statistics 2020: GLOBOCAN Estimates of Incidence and Mortality Worldwide for 36 Cancers in 185 Countries. CA Cancer J Clin. 2021; 71:209–49. 10.3322/caac.21660 33538338

[4] Keum N, Giovannucci E. Global burden of colorectal cancer: emerging trends, risk factors and prevention strategies. Nat Rev Gastroenterol Hepatol. 2019; 16:713–32. 10.1038/s41575-019-0189-8 31455888

[5] Li N, Lu B, Luo C, Cai J, Lu M, Zhang Y, Chen H, Dai M. Incidence, mortality, survival, risk factor and screening of colorectal cancer: A comparison among China, Europe, and northern America. Cancer Lett. 2021; 522:255–68. 10.1016/j.canlet.2021.09.034 34563640

[6] Mills CD. Anatomy of a discovery: m1 and m2 macrophages. Front Immunol. 2015; 6:212. 10.3389/fimmu.2015.00212 25999950 PMC4419847

[7] Zou Z, Lin H, Li M, Lin B. Tumor-associated macrophage polarization in the inflammatory tumor microenvironment. Front Oncol. 2023; 13:1103149. 10.3389/fonc.2023.1103149 36816959 PMC9934926

[8] Gao X, Long R, Qin M, Zhu W, Wei L, Dong P, Chen J, Luo J, Feng J. Gab2 promotes the growth of colorectal cancer by regulating the M2 polarization of tumor-associated macrophages. Int J Mol Med. 2024; 53:3. 10.3892/ijmm.2023.5327 37937666 PMC10688767

[9] Mahmood F, Xu R, Awan MU, Song Y, Han Q, Xia X, Zhang J. PDIA3: Structure, functions and its potential role in viral infections. Biomed Pharmacother. 2021; 143:112110. 10.1016/j.biopha.2021.112110 34474345

[10] Jordan PA, Gibbins JM. Extracellular disulfide exchange and the regulation of cellular function. Antioxid Redox Signal. 2006; 8:312–24. 10.1089/ars.2006.8.312 16677077

[11] Zou H, Wen C, Peng Z, Shao YY, Hu L, Li S, Li C, Zhou HH. P4HB and PDIA3 are associated with tumor progression and therapeutic outcome of diffuse gliomas. Oncol Rep. 2018; 39:501–10. 10.3892/or.2017.6134 29207176 PMC5783617

[12] Liu Y, Wang JX, Nie ZY, Wen Y, Jia XJ, Zhang LN, Duan HJ, Shi YH. Upregulation of ERp57 promotes clear cell renal cell carcinoma progression by initiating a STAT3/ILF3 feedback loop. J Exp Clin Cancer Res. 2019; 38:439. 10.1186/s13046-019-1453-z 31747963 PMC6864981

[13] Takata H, Kudo M, Yamamoto T, Ueda J, Ishino K, Peng WX, Wada R, Taniai N, Yoshida H, Uchida E, Naito Z. Increased expression of PDIA3 and its association with cancer cell proliferation and poor prognosis in hepatocellular carcinoma. Oncol Lett. 2016; 12:4896–904. 10.3892/ol.2016.5304 28101228 PMC5228093

[14] Germon A, Heesom KJ, Amoah R, Adams JC. Protein disulfide isomerase A3 activity promotes extracellular accumulation of proteins relevant to basal breast cancer outcomes in human MDA-MB-A231 breast cancer cells. Am J Physiol Cell Physiol. 2023; 324:C113–32. 10.1152/ajpcell.00445.2022 36374169 PMC9799142

[15] Zhang J, Li H, Li H, Lin D, Wang X, Wang K. Expression and Prognostic Significance of PDIA3 in Cervical Cancer. Int J Genomics. 2022; 2022:4382645. 10.1155/2022/4382645 36406049 PMC9674421

[16] Shimoda T, Wada R, Kure S, Ishino K, Kudo M, Ohashi R, Fujita I, Uchida E, Yoshida H, Naito Z. Expression of protein disulfide isomerase A3 and its clinicopathological association in gastric cancer. Oncol Rep. 2019; 41:2265–72. 10.3892/or.2019.6999 30720117

[17] Chiavari M, Ciotti GMP, Canonico F, Altieri F, Lacal PM, Graziani G, Navarra P, Lisi L. PDIA3 Expression in Glioblastoma Modulates Macrophage/Microglia Pro-Tumor Activation. Int J Mol Sci. 2020; 21:8214. 10.3390/ijms21218214 33153019 PMC7662700

[18] Kondo R, Ishino K, Wada R, Takata H, Peng WX, Kudo M, Kure S, Kaneya Y, Taniai N, Yoshida H, Naito Z. Downregulation of protein disulfide-isomerase A3 expression inhibits cell proliferation and induces apoptosis through STAT3 signaling in hepatocellular carcinoma. Int J Oncol. 2019; 54:1409–21. 10.3892/ijo.2019.4710 30720090

[19] Caorsi C, Niccolai E, Capello M, Vallone R, Chattaragada MS, Alushi B, Castiglione A, Ciccone G, Mautino A, Cassoni P, De Monte L, Álvarez-Fernández SM, Amedei A, et al. Protein disulfide isomerase A3-specific Th1 effector cells infiltrate colon cancer tissue of patients with circulating anti-protein disulfide isomerase A3 autoantibodies. Transl Res. 2016; 171:17–28.e1-2. 10.1016/j.trsl.2015.12.013 26772958

[20] Yang Z, Liu J, Shi Q, Chao Y, Di Y, Sun J, Zhang J, Huang L, Guo H, He C. Expression of protein disulfide isomerase A3 precursor in colorectal cancer. Onco Targets Ther. 2018; 11:4159–66. 10.2147/OTT.S154452 30050307 PMC6056171

[21] Zhang C, Yue C, Herrmann A, Song J, Egelston C, Wang T, Zhang Z, Li W, Lee H, Aftabizadeh M, Li YJ, Lee PP, Forman S, et al. STAT3 Activation-Induced Fatty Acid Oxidation in CD8+ T Effector Cells Is Critical for Obesity-Promoted Breast Tumor Growth. Cell Metab. 2020; 31:148–161.e5. 10.1016/j.cmet.2019.10.013 31761565 PMC6949402

[22] Rada M, Lazaris A, Kapelanski-Lamoureux A, Mayer TZ, Metrakos P. Tumor microenvironment conditions that favor vessel co-option in colorectal cancer liver metastases: A theoretical model. Semin Cancer Biol. 2021; 71:52–64. 10.1016/j.semcancer.2020.09.001 32920126

[23] Saeed M, Shoaib A, Kandimalla R, Javed S, Almatroudi A, Gupta R, Aqil F. Microbe-based therapies for colorectal cancer: Advantages and limitations. Semin Cancer Biol. 2022; 86:652–65. 10.1016/j.semcancer.2021.05.018 34020027

[24] Si H, Yang Q, Hu H, Ding C, Wang H, Lin X. Colorectal cancer occurrence and treatment based on changes in intestinal flora. Semin Cancer Biol. 2021; 70:3–10. 10.1016/j.semcancer.2020.05.004 32404293

[25] Weng W, Goel A. Curcumin and colorectal cancer: An update and current perspective on this natural medicine. Semin Cancer Biol. 2022; 80:73–86. 10.1016/j.semcancer.2020.02.011 32088363 PMC7438305

[26] Kennedy LB, Salama AKS. A review of cancer immunotherapy toxicity. CA Cancer J Clin. 2020; 70:86–104. 10.3322/caac.21596 31944278

[27] Ren F, Jin Q, Liu T, Ren X, Zhan Y. Proteome-wide mendelian randomization study implicates therapeutic targets in common cancers. J Transl Med. 2023; 21:646. 10.1186/s12967-023-04525-5 37735436 PMC10512580

[28] Yu F, Liu X, Li M, Liu X, Wang X, Guo M. Protein disulfide isomerase A3 as novel biomarker for endometrial cancer. Front Oncol. 2023; 13:1247446. 10.3389/fonc.2023.1247446 37909009 PMC10614013

[29] Tang X, Guo D, Yang X, Chen R, Jiang Q, Zeng Z, Li Y, Li Z. Upregulated Immunogenic Cell-Death-Associated Gene Signature Predicts Reduced Responsiveness to Immune-Checkpoint-Blockade Therapy and Poor Prognosis in High-Grade Gliomas. Cells. 2022; 11:3655. 10.3390/cells11223655 36429083 PMC9688114

[30] Zhang J, Wang K, Hainisayimu T, Li H. Pan-Cancer Analysis of PDIA3: Identifying It as a Potential Biomarker for Tumor Prognosis and Immunotherapy. Oxid Med Cell Longev. 2022; 2022:9614819. 10.1155/2022/9614819 36046686 PMC9423987

[31] Li Y, Song Z, Han Q, Zhao H, Pan Z, Lei Z, Zhang J. Targeted inhibition of STAT3 induces immunogenic cell death of hepatocellular carcinoma cells via glycolysis. Mol Oncol. 2022; 16:2861–80. 10.1002/1878-0261.13263 35665592 PMC9348600

[32] Keasey MP, Razskazovskiy V, Jia C, Peterknecht ED, Bradshaw PC, Hagg T. PDIA3 inhibits mitochondrial respiratory function in brain endothelial cells and C. elegans through STAT3 signaling and decreases survival after OGD. Cell Commun Signal. 2021; 19:119. 10.1186/s12964-021-00794-z 34922569 PMC8684072

[33] Yi B, Dai K, Yan Z, Yin Z. Circular RNA PLCE1 promotes epithelial mesenchymal transformation, glycolysis in colorectal cancer and M2 polarization of tumor-associated macrophages. Bioengineered. 2022; 13:6243–56. 10.1080/21655979.2021.2003929 35349390 PMC9208481

[34] Yang C, Dou R, Wei C, Liu K, Shi D, Zhang C, Liu Q, Wang S, Xiong B. Tumor-derived exosomal microRNA-106b-5p activates EMT-cancer cell and M2-subtype TAM interaction to facilitate CRC metastasis. Mol Ther. 2021; 29:2088–107. 10.1016/j.ymthe.2021.02.006 33571679 PMC8178444

[35] Yahaya MA, Lila MA, Ismail S, Zainol M, Afizan NA. Tumour-Associated Macrophages (TAMs) in Colon Cancer and How to Reeducate Them. J Immunol Res. 2019; 2019:2368249. 10.1155/2019/2368249 30931335 PMC6410439

